# Challenges in the management of adenocarcinoma of ampulla of Vater in pregnancy: A case report and review of literature

**DOI:** 10.1016/j.ijscr.2019.06.044

**Published:** 2019-06-26

**Authors:** Abdullah Saleh AlQattan, Feras Ahmed Alkuwaiti, Elham Saleh Alghusnah, Shoukat Ahmad Bojal, Mohammed Saad Alqahtani

**Affiliations:** a Imam Abdulrahman Bin Faisal University, Dammam, Saudi Arabia; b Department of General Surgery, King Fahad Specialist Hospital, Dammam, Saudi Arabia

**Keywords:** Ampulla of Vater, Adenocarcinoma, Jaundice, Pregnancy, Pancreaticoduodenectomy

## Abstract

**Introduction:**

Ampullary adenocarcinoma is a rare entity during pregnancy. It accounts for 0.5% of all gastrointestinal malignancies. The best treatment modality for resectable tumors with the best reported outcome is surgical resection in the form of pancreaticoduodenectomy (Whipple procedure). In this case report, we discuss the challenges in the management of ampullary adenocarcinoma in a pregnant patient.

**Case presentation:**

We report a case of 22 years old pregnant women who presented with vague abdominal pain and jaundice at the 28th week of gestation. Endoscopic retrograde cholangiopancreatography (ERCP) showed a stricture around the ampulla and distal common bile duct (CBD). The CBD was stented and a biopsy was taken. Histopathology revealed: an invasive adenocarcinoma. The patient was managed by elective pancreaticoduodenectomy after induction of vaginal delivery at the 34th week of gestation.

**Conclusion:**

Diagnosing and managing ampullary adenocarcinoma in pregnant patients in their 3rd trimester is challenging. Yet, a delayed viable delivery followed by a definitive surgery in the form of pancreaticoduodenectomy offers the best outcomes for both the mother & fetus in case of early stage disease.

## Highlights

• Ampullary adenocarcinoma is a rare entity during pregnancy.
• Diagnosing ampullary adenocarcinoma in pregnancy can be quite challenging, as the symptoms may overlap with the physiological changes of pregnancy. Furthermore, the diagnostic modalities’ invasiveness & the associated radiation exposure that might harm the fetus.
• The best treatment modality for resectable tumors is in the form of surgery (i.e.; pancreaticoduodenectomy) that is possible during pregnancy. Yet challenging as its important to choose the appropriate time of surgery to insure the viability of the fetus without risking the progression of the disease.
• Another challenge that might be encountered is intra-operatively, due to the bulkiness of the uterus which makes such a procedure even more difficult to preform safely & successfully.

## 1 Introduction

This work has been reported in line with the SCARE criteria [1].

Ampullary adenocarcinoma is a malignant tumor originating from the ampulla of Vater and it accounts for 0.5% of all gastrointestinal (GI) malignancies [2]. Ampullary adenocarcinoma is rare entity during pregnancy [3]. The best treatment modality for resectable tumors providing the best outcome is a complete surgical resection in the form of pancreaticoduodenectomy (Whipple procedure) [4]. Here, we report a case of 22 years old pregnant female at 28th week of gestation and its challenges in diagnosis and management & a review of literature.

## 2 Case presentation

**Fig. 1:**
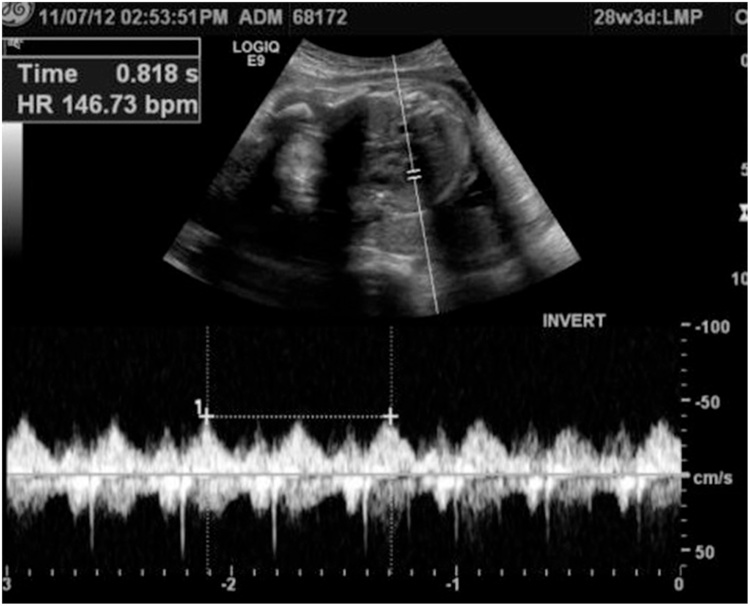
Fetal Ultrasound showed a healthy fetus.

**Fig. 2:**
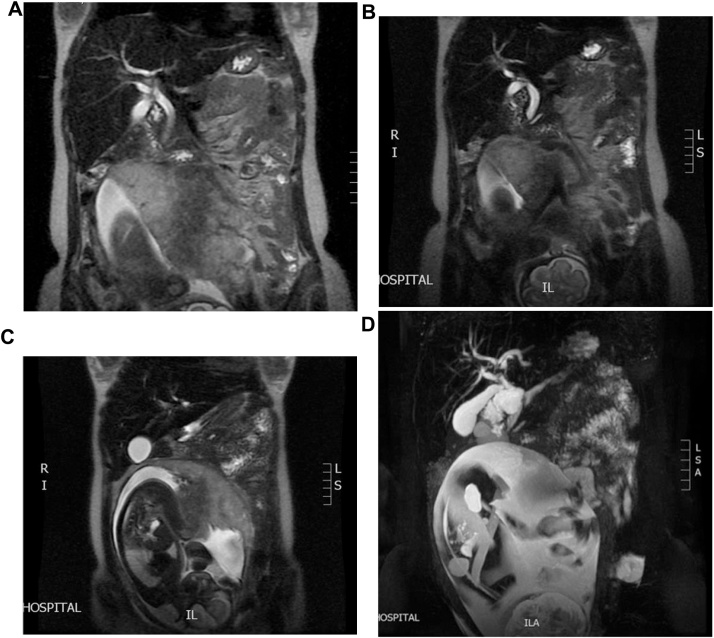
(A–D) MRCP showed dilated common CBD, pancreatic duct, and ampullary mass was 2 cm in size.

We report a case of a 22 years old pregnant female medically free, referred to us from her Obstetrician when she was complaining of abdominal pain and jaundice. Abdominal US confirmed a single intrauterine pregnancy at 28th week of gestation with appropriate growth for date and showed dilated intrahepatic ducts otherwise it was inconclusive due to the gravid uterus (Fig. 1). Her blood investigations showed a picture of cholestatic jaundice, and all other labs were within normal. So, we decided to proceed with a Magnetic resonance cholangiopancreatography (MRCP) which showed dilatation of both of the CBD (measuring 0.9 cm) & pancreatic duct, as well as an ampullary mass measuring 2 cm (Fig. 2). Later on, Endoscopic retrograde cholangiopancreatography (ERCP) with shielding of the abdomen to protect the fetus from radiation revealed an ampullary and distal CBD strictures. A punch biopsy was taken & the CBD was stented. The histopathology came as invasive adenocarcinoma & full metastatic work up was done and did not reveal any metastatic lesions. So, surgery was the best available option with the best possible outcome but we were reluctant to delay the surgery to ascertain the viability of the fetus. At 34th week of gestation induction of labor was done, both mother and the baby did well and were discharged home on 2nd day postpartum. The mother was readmitted one week later & full body CT scan repeated & there was no vascular invasion or distant metastasis.

Therefore, we proceeded with pancreaticoduodenectomy. A laparotomy incision was done, intraoperative examination of the abdomen revealed; a palpable mass at the ampulla of Vater and the stent was felt in the CBD and duodenum, a bulky uterus as the patient was still in the postpartum period. There was no vascular invasion, peritoneal deposits or any other distant metastasis. For the pancreatojejunostomy anastomosis, a two-layer end-to-side duct-to-mucosa approach was adopted. The pancreatic duct was stented to divert the pancreatic secretions away from the anastomosis. Then the hepaticojejunostomy was done in an end-to-side fashion followed by the gastrojejunostomy.

The Patient had uneventful postoperative course and was discharged 1 week after her surgery. Histopathology came as poorly differentiated invasive adenocarcinoma of ampulla of Vater with negative resection margins. Three out of thirteen lymph nodes revealed metastatic involvement so she received six cycles of adjuvant chemotherapy which she tolerated well. Upon 6 years follow up, computed tomography (CT) and positron emission tomography (PET) scans were normal with no evidence of recurrence.

## 3 Discussion

Patients with periampullary tumors can present with wide variety of gastrointestinal symptoms. The most commonly is obstructive jaundice 2ry to CBD obstruction. In other cases, they can present with biliary colic along with constitutional symptoms especially in adenocarcinoma compared to other types of GI malignancies [5].

We reviewed published articles concerning periampullary & pancreatic malignant tumors that has been diagnosed during pregnancy & confirmed by histopathology in the English literature. To the best of our knowledge, only 41 cases have been reported (Table 1) and our case is the 42nd. Porcel et al. published the first case of adenocarcinoma of ampulla of Vater in pregnant woman at 3rd trimester in 1992 [6]. Upon our review (N = 41), the common age of presentation that was found in the literature was during the 30 s in contrast to our case, where the patient presented to us at the age of 22 years old. The most common histopathological type was found to be adenocarcinoma (18 cases), followed by mucinous cystic neoplasm (16 cases), neuroendocrine neoplasms (3 cases), Solid-pseudopapillary neoplasms (3 cases) and 1 case had anaplastic.

**Table 1:** Literature review of 41 cases.

Author	Histology	Patient age	Clinical presentation	Gestation at presentation (trimester/ weeks)	Diagnosis method	Gestation for Surgery (trimester/ weeks)	Complications
Smithers et al. [12]	MCN	–	–	1st (7)	–	1 st (8)	Tumor ruptured
Baiocchi et al. [13]	MCN	–	–	3rd	–	Postpartum	None reported
Porcel et al. [6]	Adenocarcinoma	43	epigastric pain, Upper lumbar backache, nausea and vomiting.	3rd (28)	Aspiration cytology	–	severe pre-eclampsia, HELLP syndrome, left axillar venous thrombosis, Pancreatic metastasis Mother death 35 days post-partum
Olsen et al. [14]	MCN	–	–	1st (6)	–	2nd (18)	None reported
Simchuk et al. [15]	Adenocarcinoma	–	–	2nd (16)	–	2nd (16)	metastases
Sciscione et al. [16]	Neuroendocrine	37	Incidentally US finding (pancreatic mass)	2nd (19)	–	2nd (20)	Fetal death
Blackbourne et al. [17]	Adenocarcinoma	32	back pain, nausea, emesis, and dark urine	2nd (14)	Intraoperative FNA	2nd (17)	No complications
Ganepola et al. [18]	MCN	37	Abdominal pain	1st (4)	Frozen section	2nd (23)	No complications
Lopez-Tomassetti et al. [19]	MCN	26	abdominal pain and hyperemesis	2nd (20)	Histological analysis of resected mass	2nd (20)	No complications
Kato et al. [20]	MCN	33	abdominal distention	2nd (15)	MRI- Intraoperative ultrasound	2nd (20)	Pre-op IUGR
Marinoni et al. [8]	Adenocarcinoma	38	Epigastric pain	3rd (27)	Biopsy by ERCP	Postpartum	Mother death 50 days post-partum
Lin Lin Su et al. [21]	Adenocarcinoma	37	intermittent epigastric pain	2nd (22)	Ultrasound-guided liver biopsy confirmed metastatic poorly differentiated adenocarcinoma, Consistent with a pancreatic primary.	2nd	Termination of pregnancy
Ishikawa et al. [22]	MCN	33	Epigastric mass	2nd (17)	MRI	Postpartum	No complications
Al Adnani et al. [23]	Adenocarcinoma	27	Prenatal care: HTN, fallen fetal growth	3rd (30)	Pancreatic biopsy	–	IUGR, placental metastasis, Mother death 3 moths postpartum
Herring et al. [24]	MCN	34	Accidental finding of abdominal mass during routine follow up	1st (3)	Histological analysis of resected mass	2nd (17)	No complications
Ozden et al. [25]	MCN	32	Epigastric pain	3rd (36)	Histological analysis of resected mass	3rd (36)	Tumor rupture
Wiseman et al. [26]	MCN	32	LUQ pain with palpable mass	2nd (15)	MRI- US guided drainage	2nd (16)	Pre-op Intractable nausea
Hakamada et al. [27]	Anaplastic	38	Incidentally US finding	1st trimester, 1st pregnancy	MRI- US	2nd trimester, 2nd pregnancy	Intractable nausea, upper GI bleed (pre-op), local recurrence (post-op)
Ikuta et al. [28]	MCN	30	Left hypochondrial pain	1st (10)	CT, - US guided drainage	Post-abortion	Missed abortion
Kamphues et al. [29]	Neuroendocrine	32	Arterial HTN	2nd (19)	US, MRI	2nd (19)	Splenic vein thrombosis renal artery compression (intra-op)
Kamphues et al. [29]	Neuroendocrine	35	Vomiting, weight loss	2nd (16)	Needle biopsy	2nd (18)	No complications
Kakoza et al. [11]	Adenocarcinoma	40	Epigastric pain, nausea, vomiting	2nd (24)	Duodenal mucosal biopsy	Postpartum	Liver metastasis, mother death 6 months post-op
Asciutti et al. [30]	MCN		Epigastric pain + mass	2nd (23)	–	Postpartum	Pancreatitis
Onuma et al. [31]	Adenocarcinoma	32	frequent uterine contractions	3rd (30)	CT, Histological analysis of resected mass	3rd (34)	Pre-op Gastric perforation
Naganuma et al. [32]	MCN	32	threat of premature labor	3rd (33)	CT, Histological analysis of resected mass	3rd (34)	Tumor rupture, local recurrence 6 months pos-op
Perera et al. [7]	Adenocarcinoma	25	Epigastric pain, nausea, One episode of emesis	2nd (20)	MRCP, ERCP	No surgery, underwent chemotherapy	Mother Death
Lubner et al. [33]	Adenocarcinoma	37	nausea, vomiting, back pain, acholia, and dark colored urine	2nd (16)	EUS with FNA	2nd (18)	Death 12 months post diagnosis
Marci et al. [34]	Adenocarcinoma	36	Epigastric pain, vomiting, weight loss	3rd (35)	US, CT	Postpartum	(PRE-OP) Acute renal failure
Feng et al. [35]	SPNs	26	Incidentally abdominal mass by US	2nd (14)	US, MRI, needle biopsy	2nd (14)	3rd postoperative day pancreatic fistula occurred
Boyd et al. [36]	MCN	21	abdominal distention and fullness	1st (10)	CT, MRI	2nd (20)	No complications
Boyd et al. [36]	Adenocarcinoma	29	Emesis, epigastric pain	3rd (37)	US, CT	Postpartum	2 weeks post-op PE, iliac & femoral veins thrombosis, DIC, multiorgan system failure & death.
Boyd et al. [36]	Adenocarcinoma	37	right upper back pain, nausea and vomiting, alcoholic stools, and dark urine	2nd (17)	US, ERCP, FNA	2nd (19)	4 months post-op liver metastasis, 1-year post-op death.
Liu et al. [37]	Adenocarcinoma	31	weight loss and progressive, positional dyspnea	3rd trimester	US, CT, PET scan	3rd (34)	Ovarian cyst rupture, (post-op) Pleural effusion, ascites
Tsuda et al. [38]	MCN	28	Referred as case of abdominal tumor	1st (9)	US, MRI,	2nd (18)	Mild glucose intolerance post-op.
Tica et al. [39]	MCN	27	abnormal sonogram	3rd (29)	MRI	postpartum	No complications
Huang et al. [40]	SPNs	29	Epigastric pain, backache, nausea, and vomiting	2nd (19)	US, MRI	2nd (19)	Tumor rupture
MacDonald et al. [41]	SPNs	23	Incidentally abdominal mass by US	2nd (14)	US, MRI	2nd (18)	No complications
Labarca-Acosta et al. [42]	Adenocarcinoma	35	Vomiting, pain in the left epigastrium and hypochondrium, general weakness and weight loss.	16 weeks	Fine-needle biopsy	–	Maternal death
Aker et al. [43]	Adenocarcinoma	27	Right upper quadrant pain, nausea,and vomiting.	2nd (26)	ascites cytology	Post-partum	Fetal death Metastasis to placenta, hepatic, supraclavicular lymph node
Davis et al. [44]	Adenocarcinoma	34	abdominal pain and failure to gain weight appropriately in pregnancy.	2nd (26)	Pathology on fine-needle aspiration of the pancreatic head mass confirmed pancreatic adenocarcinoma	–	Death 4 months after C-section
Aynioglu et al. [45]	Adenocarcinoma	36	recurrent severe abdominal pain radiating to the back, jaundice, nausea, and vomiting.	3rd (28)	Histopathological analysis	Post-partum	No complication

MCN = mucinous cystic neoplasm; HELLP = hemolysis& elevated liver enzymes level and low platelet; IUGR = intrauterine growth restriction; SPNs = Solid-pseudo papillary neoplasms.

In case of pregnancy, diagnosing of ampullary adenocarcinoma can be challenging. The way it does that is by making alarming symptoms of malignancy through the physiological changes of pregnancy. As jaundice can develop frequently in the 3rd trimester due to benign intrahepatic cholestasis, which can be misleading initially as in our case. Furthermore, other symptoms that adenocarcinoma of ampulla of Vater cause like abdominal discomfort, nausea & vomiting also can happen in normal pregnancy. Another challenge is that the gravid uterus that decreases the sensitivity of non-invasive imaging modalities [5]. The reported cases demonstrate a variety of gestational ages at presentation, which indicated a clinical difficulty in diagnosis. Unlike our case, most of the reported cases presented at 2nd trimester.

There are different radiological modalities that can aid in obtaining a confirmatory diagnosis and staging of ampullary carcinoma but not all of these can be used freely during pregnancy such as ERCP or CT scan, due to the risk of radiation exposure to the fetus [7]. The main diagnostic modalities for pregnant women are ultrasonography and MRCP. The ultrasonography is mainly used to identify the presence of biliary dilation & obstruction while MRCP can visualize the mass [8].

In our case, the MRCP revealed an ampullary mass measuring 2 cm with no vascular involvement. ERCP is used during pregnancy for diagnosis by obtaining a biopsy, stenting for biliary draining and to prepare the patient for surgery or if the tumor is not operable. To minimize the risk radiation exposure to the fetus the patient should have a lead shield in place as we did in our case. Biochemically, an elevated carbohydrate antigen 19-9 (CA 19-9) level may help to guide the diagnosis towards an ampullary adenocarcinoma [4]. Nevertheless, US and MRCP were the main diagnostic modalities reported in the literature.

Management wise, surgical resection is usually done by a Whipple procedure (Pancreaticoduodenectomy) followed by an adjuvant therapy is the standard of care for early stage disease. Such surgery carries a risk of many complications. One important complication to consider is pancreatic fistula or leak with a reported incidence ranging from 2 to 40% [9].

To decrease the risk of such complication, intraoperatively a pancreatic duct stent can be used. Adaptation of this technique to protect the anastomotic site & decrease the chance of any leak have led good to outcome of such procedure in some centers [10].

Surgical intervention is challenging in pregnant patients, the appropriate time to intervene depends on the gestational age at the time of diagnosis and the stage of the disease. The challenges that might be encountered in first trimester, is the risk of spontaneous abortion and the best strategy is to abort the pregnancy. While in the second trimester induced delivery can’t be applied as gestational age is not compatible with life and it is the most dangerous time to put the patient on chemotherapy. In the third trimester, the large size of the uterus is another challenge especially intraoperatively [11].

In our patient, the obstetrician induced labor at week 34 of gestation & Whipple procedure was done afterwards and the major challenge was a presence of a bulky uterus during the surgery.

## 4 Conclusion

Obtaining a diagnosis of ampullary adenocarcinoma in pregnant patients in their third trimester & managing them can be challenging due to; 1) the overlapping symptoms between it & the physiological changes of pregnancy, 2) the limitation of using the appropriate diagnostic modalities in order to avoid radiation exposure to the fetus, 3) the intraoperative technical difficulties 2ry to the gravid uterus. Yet, a delayed viable delivery followed by a definitive surgery in the form of pancreaticoduodenectomy is still achievable with a multi-disciplinary approach & good perioperative preparation in early stage disease.

## Conflicts of interest

None.

## Funding

This research did not receive any specific grant from funding agencies in the public, commercial, or not-for-profit sectors.

## Ethical approval

Case reports are exempted from ethical approval according to our institution policies.

## Consent

Written informed consent was obtained from the patient for publication of this case report.

## Author contribution

Abdullah Saleh AlQattan: study design, data collection, writing the paper, reviewing and editing the case report.

Feras Ahmed Alkuwaiti: study design, data collection, writing the paper.

Elham Saleh Alghusnah: study concept, reviewing article, correction and editing of the case report.

Shoukat Ahmad Bojal: study concept, reviewing article, correction and editing of the case report.

Mohammed Saad Alqahtani: study concept, reviewing the final manuscript of the case report, final approval.

## Registration of research studies

N/A.

## Guarantor

Dr. Mohammed Saad Alqahtani.

## Provenance and peer review

Not commissioned, externally peer-reviewed.
